# JMJD2C promotes colorectal cancer metastasis via regulating histone methylation of MALAT1 promoter and enhancing β-catenin signaling pathway

**DOI:** 10.1186/s13046-019-1439-x

**Published:** 2019-10-29

**Authors:** Xinnan Wu, Ruixiao Li, Qing Song, Chengcheng Zhang, Ru Jia, Zhifen Han, Lihong Zhou, Hua Sui, Xuan Liu, Huirong Zhu, Liu Yang, Yan Wang, Qing Ji, Qi Li

**Affiliations:** 1 Department of Medical Oncology and Cancer Institute of Medicine, Shuguang Hospital, Shanghai University of Traditional Chinese Medicine, Shanghai, China; 2 Department of Medical Oncology, Suzhou TCM Hospital Affiliated to Nanjing University of Chinese Medicine, Suzhou, China; 3 Department of Medical Oncology, Longhua Hospital, Shanghai University of Traditional Chinese Medicine, Shanghai, China; 4 Academy of Integrative Medicine, Shanghai University of Traditional Chinese Medicine, Shanghai, China

**Keywords:** Histone demethylase JMJD2C, MALAT1, β-Catenin signaling pathway, Colorectal cancer, Metastasis

## Abstract

**Background:**

Our previous work demonstrated that lncRNA-MALAT1 was overexpressed in recurrent colorectal cancer (CRC) and metastatic sites in post-surgical patients. However, the upstream regulatory mechanism of MALAT1 is not well-defined. Histone demethylase JMJD2C holds great potential of epigenetic regulating mechanism in tumor diseases, especially the moderating effect on the promoter activity of targeted genes associated closely with tumor development. Therefore, we herein investigated whether JMJD2C could epigeneticly regulate the promoter activity of MALAT1 and the downstream β-catenin signaling pathway, thereby affecting the metastatic abilities of CRC cells.

**Methods:**

JMJD2C expressions in human CRC samples were detected by real-time PCR and immunohistochemistry staining. Gene silencing and overexpressing efficiencies of JMJD2C were confirmed by real-time PCR and western blot. The migration of CRC cells in vitro were tested by transwell and wound healing assays. The protein expression and cellular localization of JMJD2C and β-catenin were characterized by immunofluorescence staining and western blot. The histone methylation level of MALAT1 promoter region (H3K9me3 and H3K36me3) was tested by ChIP-PCR assays. The promoter activity of MALAT1 was detected by luciferase reporter assay. The expressions of MALAT1 and the downstream β-catenin signaling pathway related genes in CRC cells were detected by real-time PCR and western blot, respectively. The nude mice tail vein metastasis model was established to observe the effect of JMJD2C on the lung metastasis of CRC cells in vivo.

**Results:**

Our present results indicated that histone demethylase JMJD2C was overexpressed in matched CRC tumor tissues of primary and metastatic foci, and CRC patients with lower JMJD2C expression in primary tumors had better prognosis with longer OS (Overall Survival). The following biological function observation suggested that JMJD2C promoted CRC metastasis in vitro and in vivo. Further molecular mechanism investigation demonstrated that JMJD2C protein translocated into the nuclear, lowered the histone methylation level of MALAT1 promoter in the sites of H3K9me3 and H3K36me3, up-regulated the expression of MALAT1, and enhanced the β-catenin signaling pathway in CRC cells.

**Conclusion:**

Our data demonstrated that JMJD2C could enhance the metastatic abilities of CRC cells in vitro and in vivo by regulating the histone methylation level of MALAT1 promoter, thereby up-regulating the expression of MALAT1 and enhancing the activity of β-catenin signaling pathway, providing that JMJD2C might be a novel therapeutic target for CRC metastasis.

## Background

Colorectal cancer (CRC) is one of the most common causes of cancer death, bringing much inconvenience to people’s daily life [1]. Although an increasing number of new therapy methods are applied to CRC treatment, the therapeutic efficacy for advanced CRC patients is not good. Tumor recurrence or metastasis is still the main cause of therapy failure [2] and poor prognosis. The 5-year survival rates of CRC patients were about 90% for the ones with only primary tumors, 70.4% for the ones with lymph node or peripheral metastasis, and 12.5% for the ones with distal metastasis [3].

Long non-coding RNAs (lncRNAs) have been discovered to act important roles in tumor development [4]. Metastasis associated in lung adenocarcinoma transcript 1 (MALAT1) is one of these functional lncRNAs, which is highly expressed in CRC patients. In our previous studies, we have successfully demonstrated that MALAT1 could promote tumor growth and metastasis in CRC by binding to SFPQ (PTB-associated splicing factor) and releasing oncogene PTBP-2 (polypyrimidine tract binding protein) from the SFPQ/PTBP-2 complex [5], and increase the nuclear translocation of β-catenin from cytoplasm, thereby activating downstream genes of β-catenin signaling pathway [6, 7]. Ying et al also revealed that, in bladder cancer, MALAT1 promoted EMT by activating Wnt signaling in vitro [8]. However, the upstream regulatory mechanism of MALAT1 is not well-elucidated.

Jumonji domain-containing protein 2C (JMJD2C), also known as KDM4C, could modulate transcription factors, establish global chromatin environments and regulate gene expression [9–11]. A number of evidences have suggested the association between JMJD2C protein and various tumors [12–15]. Histone demethylase JMJD2C holds great potential of epigenetic regulating mechanism in tumor diseases [16, 17], especially the regulating effect on the promoter activity of targeted genes. Based on bioinformatics analysis of the MALAT1 promoter and our previous studies, we hypothesized that, JMJD2C might influence MALAT1 promoter activity, thereby activating MALAT1/β-catenin signaling pathway and leading to the promotion of CRC metastasis.

Therefore, in this study, we aimed to investigate the new effect of JMJD2C on the metastasis of CRC cells in vitro and in vivo, and to detect the regulatory effect of JMJD2C on the expression of MALAT1 and β-catenin signaling pathway related genes. Most importantly, the potential epigenetic regulating mechanism of JMJD2C on MALAT1 was the focus of this research.

## Materials and methods

### Human tissues collection and cell culture

A total of 78 human primary CRC tissues without metastasis and 46 human primary CRC tissues with matched hepatic or lung metastasis were collected between 2006 and 2015 at Shuguang Hospital, Shanghai University of Traditional Chinese Medicine, and Fudan University Shanghai Cancer Center. HCT116 (human colon, CRC) and LoVo (human colon, Dukes’ type C, grade IV, colorectal adenocarcinoma) cells were purchased from the ATCC (Manassas, USA), and cultured in RPMI-1640 and F12K medium, respectively. Both mediums were supplemented with 10% heat-inactivated FBS, 100 U ml^− 1^ penicillin, and 100 μg ml^− 1^ streptomycin. These cells were incubated under 37 °C, 5% CO_2_ conditions. All the experimental procedures were approved by the Institutional Review Board of Shuguang Hospital, Shanghai University of Traditional Chinese Medicine.

### Plasmids construction and lentivirus infection

Three shRNA fragments for human JMJD2C (Gene ID:23081) were synthesized by the Sangon Biotech company (Shanghai, China), sub-cloned into the pLKD shRNA vector, and named pLKD-CMV-G&PR-U6-JMJD2C-shRNA1/2/3, respectively. The full length of JMJD2C gene was amplified by PCR, following by gene sequencing, and the right full fragments was cloned into the plenti overexpression vector, named plenti-CMV-EGFP-P2A-JMJD2C-3FLAG. These above plasmids were used to transfect well-growing state HCT116 and LoVo cells by using Lipofectamine 3000 transfection reagent. After 48 h transfection, the transfected cells were selected with neomycin (G418, 1 mg ml^− 1^, Sigma, USA). Recombinant lentiviruses containing pLV4-shRNA/NT, pLV4-shRNA/JMJD2C, pLV4-empty vector, and pLV4-JMJD2C^+/+^ were prepared by GeneChem (Shanghai, China), respectively. HCT116 or LoVo cells were infected with 2 × 10^6^ transducing units of corresponding lentiviruses, and were selected with 2 μg/ml puromycin for 2 weeks. The efficiencies of knockdown or overexpression of JMJD2C were determined by real-time PCR and western blot.

### Real-time PCR

Total RNA was extracted using the TRIzol reagent (Takara) according to the manufacturer’s instructions. The RNA concentrations were determined using a NanoDrop ND-1000 (NanoDrop). cDNA was synthesized with the PrimeScript RT Reagent Kit (TaKaRa) using 500 ng total RNA as template. Real-time PCR analyses were conducted to quantitate KDM4C mRNA and lncRNA-MALAT1 relative expression using SYBR Premix Ex Taq (TaKaRa) with GAPDH as an internal control. The Real-time PCR results were defined from the threshold cycle (Ct), and relative expression levels were calculated by using the 2^-△△Ct^ method. PCR was performed using an ABI 7500 instrument (Applied Biosystems, USA). The primers used for real-time PCR analysis were as follow: KDM4C, forward primer: 5-AGGCCTAAGGCTGATGAGGA-3, reverse primer: 5-TTGGCCATGAAAGCTCGGAT-3; MALAT1, forward primer: 5-GCTCTGTGGTGTGGGATTGA-3, reverse primer: 5-GTGGCAAAATGGCGGACTTT-3, GAPDH, forward primer: 5-GGTGGTCTCCTCTGACTTCAACA-3, reverse primer: 5-CCAAATTCGTTGTCATACCAGGAAATG-3.

### Luciferase reporter assay

To test MALAT1 promoter activity, HCT116 or LoVo cells were co-transfected with the recombinant plasmid pGL3-basic-MALAT1 promoter with a control positive plasmid pRL-SV40 as previously described [18]. The promoter activity was analyzed using a commercial dual-luciferase assay kit (Promega, USA) according to the manufacturer’s instructions.

### Immunofluorescence staining

To observe the expression and location of JMJD2C and β-catenin, HCT116 and LoVo cells after transfection were plated at a density of 2.0 × 10^4^/ml in 6-well plates, fixed with methanol, blocked with 5% BSA. The cells were first stained with β-catenin mouse antibody followed by Cy3-conjugated goat anti-mouse IgG (Millipore). After the cells were washed four times with PBS, the JMJD2C rabbit antibody was added, followed by FITC-conjugated goat anti-rabbit IgG (Millipore). Nuclear staining was done with 4′,6-diamidino-2-phenylindole dihydrochloride (DAPI) solution. Cells were imaged using TCS SP2 spectral confocal system (Leica, Germany). All experiments were conducted according to instructions from the antibody manufacturer.

### Western blot analysis

Protein lysates from cells were prepared in lysis buffer and centrifuged at 12000 rpm at 4 °C. The primary antibodies used were JMJD2C (Santa Cruz, USA), β-catenin (CST, USA), c-Myc (CST, USA), ITGBL1 (Abcam, USA), PCNA (CST, USA) and GAPDH (CST, USA). The secondary antibody used was HRP-labeled goat anti-rabbit/mouse IgG (H + L) (Beyotime, China). Each band was quantitatively analyzed using quantity one Software and normalized to the expression of GAPDH in the same lane.

### Chromatin immunoprecipitation assay

ChIP assays were performed using a ChIP assay kit (Millipore, USA). Chromatin extracts were immunoprecipitated using 10 μg anti-JMJD2C (Abcam, USA), 4 μg anti-H3K9 (Abcam, USA), and 4 μg anti-H3K36 (Abcam, USA), respectively. IgG (Merck, Germany) were used as mock ChIP controls. Fold enrichments were calculated by determining the ratios of the amount of immunoprecipitated DNA to that of the input sample, and were normalized to the level observed at a control region, which was defined as 1.0. The ChIP primers for MALAT1 promoter are as follow: forward primer: 5-GGTCAGCCTGAGACCACTTC-3, reverse primer: 5-CTGTGCCTGTTCTGGGGAAT-3.

### Transwell assay

To measure cell migration, the transfected HCT116 and LoVo cells (2 × 10^5^) were seeded to the upper chambers and cultured with 100 μl serum-free F12K or RPMI1640 medium, whereas the lower chamber was filled with 600 μl F12K or RPMI1640 medium containing 15% FBS and 10 μg ml^− 1^ fibronectin. After incubation for 48 h at 37 °C and 5% CO_2_, the chambers were fixed with 4% paraformaldehyde and stained with 0.1% crystal violet. The cells in the upper chamber were carefully removed with a cotton swab. The numbers of cells were analyzed by counting five independent visual fields under a DMI3000 B inverted microscope (Leica, USA) with a 20 × objective. All assays were performed in triplicate and independently repeated three times.

### Wound healing assay

Cells (4.5 × 10^5^) were seeded on a six-well plate to form a confluent monolayer in 10% FBS-containing medium. The monolayer cells were scratched by a plastic tip and washed with PBS to remove cell debris; 0.5% FBS-containing F12K or RPMI1640 were then added to each well, and the scratched monolayer was incubated in a 37 °C incubator with 5% CO_2_ for 24 h. Wound closure was measured in five random fields at × 200 magnification using Image J software and a DMI3000B inverted microscope (Leica, USA). Percentage of wound healing was calculated as follows: migrated cell surface area/ total surface area × 100, in which, migrated cell surface area = length of cell migration (mm) × 2 × length of defined areas, total surface area = beginning width × length of defined areas.

### MTT assay

Cells were cultured at a density of 2.5 × 10^3^ cells per well in a flat-bottomed 96-well plate. MTT [3-(4,5)-dimethylthiahiazo(−z-y1)-3,5-di-phenytetrazoliumromide] was used to determine the cell viability by measuring the absorbance at 490 nm. All assays were performed in triplicate and independently repeated three times.

### Flow cytometry

Cells were harvested by trypsinization, washed once with cold PBS. Then, the cells were stained with PI (propidium iodide) and anti-Annexin-V antibody (Becton Dickinson, USA) at 4 °C for 1 h. Subsequently, cells were washed once with PBS and analyzed by FACS (BD, USA).

### In vivo analysis

All the animal experiments were performed under the approval of the Institutional Committee for Animal Research and were in accordance with national guidelines for the care and use of laboratory animals. HCT116-shRNA/NT, HCT116-shRNA/JMJD2C, HCT116-empty vector, and HCT116-JMJD2C^+/+^ cells (2 × 10^6^ cells in 100 μl) were injected into female BALB/c nude mice (6–8 weeks old, obtained from SLAC Laboratory Lab, Shanghai, China) by tail intravenous to establish the lung metastasis mice model, respectively. Mice were euthanized 42 days post injection, and the lung metastases images were observed and quantified by LB983 NIGHTOWL II system. The numbers of lung metastatic nodes were measured, and the metastatic tumor tissues were collected for immunohistological and western blot analysis with JMJD2C, β-catenin, c-Myc, and ITGBL1 antibody.

### HE staining and IHC staining

Paraffin-embedded tissues were sectioned for HE (hematoxylin and eosin) and IHC (immunohistochemistry) staining. For IHC analysis, the experiments were performed using the first antibody, HRP-conjugated secondary antibody, and DAB (diaminobenzidine) detection reagents. The DMI3000B microscope connected to the digital imaging system was applied for taking photographs and the following analysis. All the data were evaluated and classified blindly by two investigators (ZYW and LLW) from the pathology department of Shuguang Hospital, Shanghai University of Traditional Chinese Medicine.

### Statistical analysis

All data are presented as means with standard deviation (SD) or median with 95% confidence interval (95% CI). Statistic comparison was performed using the Student’s t-test, one-way ANOVA analysis, Mann-Whitney test, or Kruskal-Wallis test, as appropriate, with the significance level at *P*<0.05. All statistical analyses were completed with SPSS 22 software.

## Results

### Overexpression of JMJD2C in CRC metastatic foci predicted poor prognosis

**Fig. 1:**
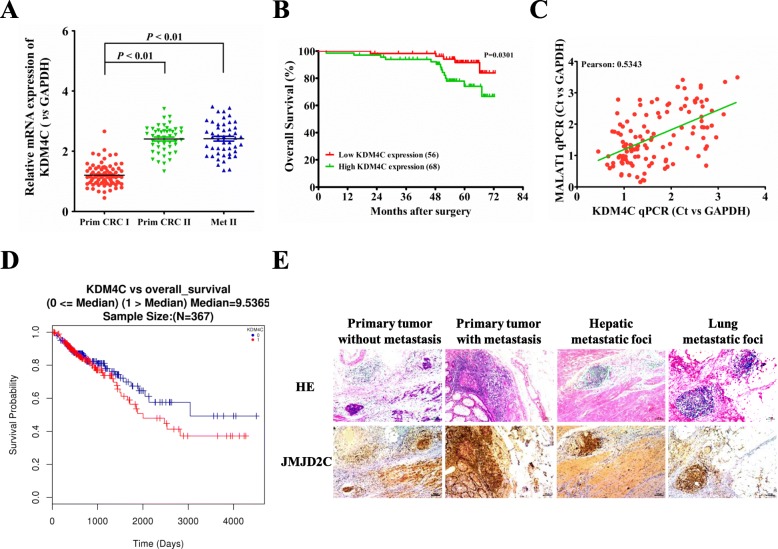
KDM4C expression in post-surgical, recurrent primary and metastatic sites, comparing to primary sites of non-recurrent CRC patients. **a** Expression levels of KDM4C mRNA in 124 CRC tissues and matched metastatic sites were analyzed by real-time PCR. The significant differences between primary tumor I (without paired metastatic tissues) and primary tumor II (with paired metastatic tissues, Metastasis II) were analyzed using the Wilcoxon signed-rank test. **b** Kaplan-Meier analyses of the correlations between KDM4C mRNA expression levels and overall survival (OS) of 124 CRC patients, and the median expression level was used as the cutoff. **c** The co-expression between KDM4C and MALAT1was showed in 124 cases from our datasets. **d** The mRNA expression of JMJD2C in 367 CRC primary tissues from TCGA dataset, and its association with CRC prognosis, including OS. **e** Immunohistochemical analysis of JMJD2C protein (encoded by KDM4C) in representative CRC and metastatic lung/liver tissues, including primary CRC tumor without paired metastatic tissues, and primary CRC tumor with paired metastatic tissues (scale bars, 100 μm, respectively). *, *P* < 0.05; **, *P* < 0.01 (*t* test)

Firstly, the expression levels of KDM4C mRNA (encoding JMJD2C protein) were detected in 124 CRC tumor specimens. The results demonstrated that, the levels of KDM4C mRNA were significantly higher in recurrent tumors and metastatic sites than that of non-recurrent ones (Fig. 1a), and the mRNA levels were closely associated with TMN stages and distant metastasis (Table 1). Survival analysis concluded that CRC patients with lower KDM4C mRNA expression had longer OS (Overall Survival) (Fig. 1b). In our previous reports, we have shown in 124 CRC tumor specimens that MALAT1 had higher expression levels in recurrent primary and metastatic sites, relative to non-recurrent primary tumors (7). In the present study, we further found that, highly matched co-expression of KDM4C mRNA and MALAT1 was found in our 124 CRC cases (Fig. 1c). In addition, a TCGA (The Cancer Genome Atlas)-dataset analysis also showed the elevated mRNA levels of KDM4C in 367 CRC primary tissues, and the KDM4C mRNA expression was inversely correlated with OS of CRC patients (Fig. 1d). Next, the immunohistochemistry staining further revealed significantly higher protein levels of JMJD2C (encoded by KDM4C) in recurrent tumors and lung/liver metastasis than the ones in non-recurrent tumors (Fig. 1e), implying that JMJD2C overexpression correlated closely with CRC progression.

**Table 1:** Association between KDM4C expression and clinicopathological variables of CRC patients

Variables	Low KDM4C expression (*n* = 67)	High KDM4C expression (*n* = 57)	*P*
	n (%)	n (%)	
Age			
> 65	13 (19.40)	10 (17.54)	0.8210
≤ 65	54 (80.60)	47 (82.46)	
Gender			
Male	39 (58.21)	39 (68.42)	0.2673
Female	28 (41.79)	18 (31.58)	
Tumor site			
Rectum	37 (55.22)	36 (63.16)	0.4642
Colon	30 (44.78)	21 (36.84)	
Tumor differentiation			
Well	20 (29.85)	19 (33.33)	0.7019
Moderate + Poor	47 (70.15)	38 (66.67)	
TNM stage			
Stage II	35 (52.23)	12 (21.05)	0.0004**
Stage III	32 (47.77)	45 (78.95)	
Lymph vascular invasion			
Positive	44 (65.67)	42 (73.68)	0.4347
Negative	23 (34.33)	15 (26.32)	
Perineural invasion			
Positive	52 (77.61)	46 (80.70)	0.8253
Negative	15 (22.39)	11 (19.30)	
Liver metastasis			
Positive	9 (13.43)	22 (38.60)	0.0017**
Negative	58 (86.57)	35 (51.40)	
Lung metastasis			
Positive	1 (1.49)	8 (14.04)	0.0114*
Negative	66 (98.51)	49 (85.96)	

*, *P* < 0.05; **, *P* < 0.01

### JMJD2C promoted the migration of CRC cell lines in vitro

**Fig. 2:**
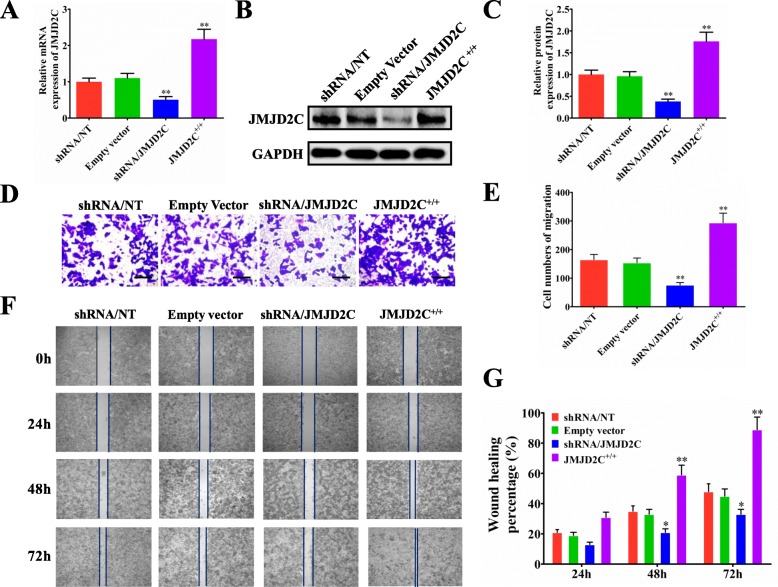
JMJD2C promoted the metastasis of CRC cells in vitro. **a-c** Real time PCR and western blotting were performed to confirm the gene silencing and overexpressing efficiency for JMJD2C. HCT116 was transiently transfected with shRNA/NT vector, shRNA/JMJD2C vector, empty overexpression vector, or JMJD2C overexpression vector. **d** Migration assays of HCT116 cells transfected with shRNA/NT, shRNA/JMJD2C, empty vector, or JMJD2C overexpression vector, respectively. **e** Numbers of migrated cells are shown as mean ± SD; *n* = 3. **f-g** Wound healing assay was used to evaluate the effect of JMJD2C on migration of HCT116 cells. *, *P* < 0.05; **, *P* < 0.01 (*t* test)

To thoroughly understand the biological function of JMJD2C, we first knocked down and overexpressed JMJD2C expression using shRNA and overexpressing vector, respectively. Real-time PCR assay was carried out to detect the mRNA expression of JMJD2C and showed efficient down-regulation or up-regulation of JMJD2C in HCT116 and LoVo cells (Fig. 2a, Additional file 1: Figure S1A). The protein expression of JMJD2C was further confirmed by western blot and quantitative assay (Fig. 2b, c, Additional file 1: Figure S1B, C). Subsequently, by transwell and wound healing assays in HCT116 and LoVo cells, we found a positive role of JMJD2C in promoting CRC cells migration (Fig. 2d-g, Additional file 1: Figure S1D, E). Additionally, we also found that, JMJD2C could promote the proliferation of CRC cells (Additional file 2: Figure S2A), but affected little on the apoptosis of CRC cells (Additional file 2: Figure S2B).

### Nuclear translocation of JMJD2C lowered the histone methylation level of MALAT1 promoter in CRC

**Fig. 3:**
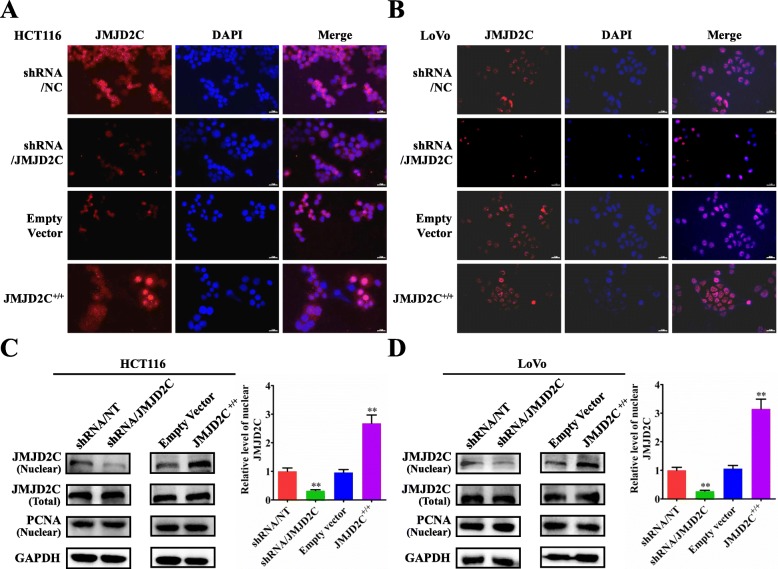
Translocation of JMJD2C protein from the cytoplasm into the nuclei in CRC cells in vitro. **a**-**b** Immunofluorescence detection of JMJD2C protein in HCT116 or LoVo cells transiently transfected with shRNA/NT vector, shRNA/JMJD2C vector, empty overexpression vector, or JMJD2C overexpression vector. **c**-**d** Western blot and quantitative assay of JMJD2C protein (nuclear and whole cell lysates) in HCT116 or LoVo cells transiently transfected with shRNA/NT vector, shRNA/JMJD2C vector, empty overexpression vector, or JMJD2C overexpression vector. *, *P* < 0.05; **, *P* < 0.01 (*t* test)

Histone demethylase JMJD2C holds great potential of epigenetic regulating mechanism in tumor diseases [19–27], especially its important regulating effect on the promoter activity of targeted genes [28, 29]. By immunofluorescent staining assay, we found that, knockdown of JMJD2C could significantly decrease the nuclear accumulation of JMJD2C protein in CRC cells, while overexpression of JMJD2C could effectively elevate the distribution of JMJD2C protein in the nuclei of CRC cells (Fig. 3a, b). Then, above results were further validated by the western blot detection (Fig. 3c, d).

**Fig. 4:**
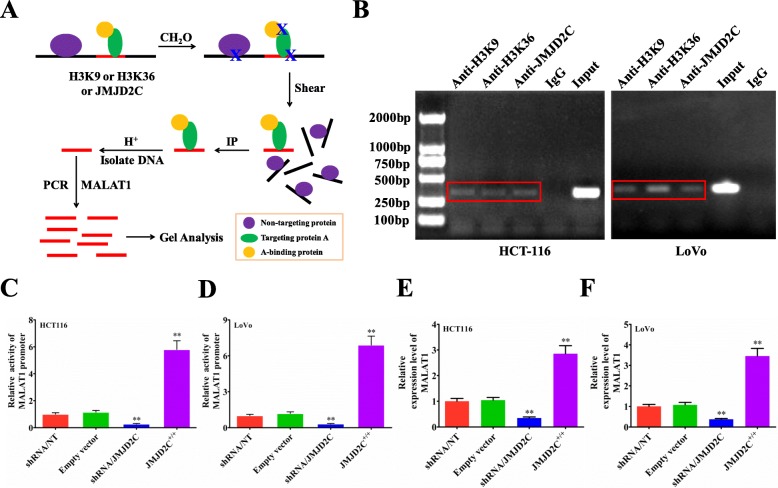
JMJD2C promoted MALAT1 expression by regulating the histone methylation level of MALAT1 promoter. **a** Schematic diagram of the ChIP (Chromatin Immunoprecipitation) procedure for detecting the effect of JMJD2C on the histone methylation level of MALAT1 promoter in sites of H3K9m3 and H3K36m3. Purple ovals: no-targeting protein; Yellow ovals: targeting protein A; Green ovals: protein binding to targeting protein A. **b** JMJD2C, H3K9, H3K36 antibody and MALAT1 special primers were used to investigate the interaction between JMJD2C and MALAT1 promoter, IgG was used as the negative control. **c-d** MALAT1 promoter activities assay in HCT116 or LoVo cells transiently transfected with shRNA/NT vector, shRNA/JMJD2C vector, empty overexpression vector, or JMJD2C overexpression vector. **e-f** Real time PCR assay of MALAT1 levels in HCT116 or LoVo cells transiently transfected with shRNA/NT vector, shRNA/JMJD2C vector, empty overexpression vector, or JMJD2C overexpression vector. *, *P* < 0.05; **, *P* < 0.01 (*t* test)

Urged by above data, we next studied if nuclear JMJD2C could bind to the promoter of MALAT1 to affect the expression of MALAT1 by chromatin immunoprecipitation (ChIP) (Fig. 4a). Based on our ChIP assay, we found that JMJD2C could bind to the promoter of MALAT1, and regulate lower the histone methylation level of MALAT1 promoter in sites of H3K9m3 and H3K36m3, whether not only in HCT116 or but also in LoVo cells (Fig. 4b). Next, the luciferase reporter assay further confirmed that, JMJD2C could promote the promoter activity of MALAT1 gene (Fig. 4c, d). Moreover, the real-time PCR results also demonstrated that, JMJD2C could promote the transcriptional level of MALAT1 (Fig. 4e, f). All above results suggested that JMJD2C could translocate into the nuclei, regulate lower the histone methylation level of MALAT1 promoter, and elevate the expression of MALAT1.

### JMJD2C enhanced nuclear translocation of β-catenin and activated β-catenin signaling in CRC

**Fig. 5:**
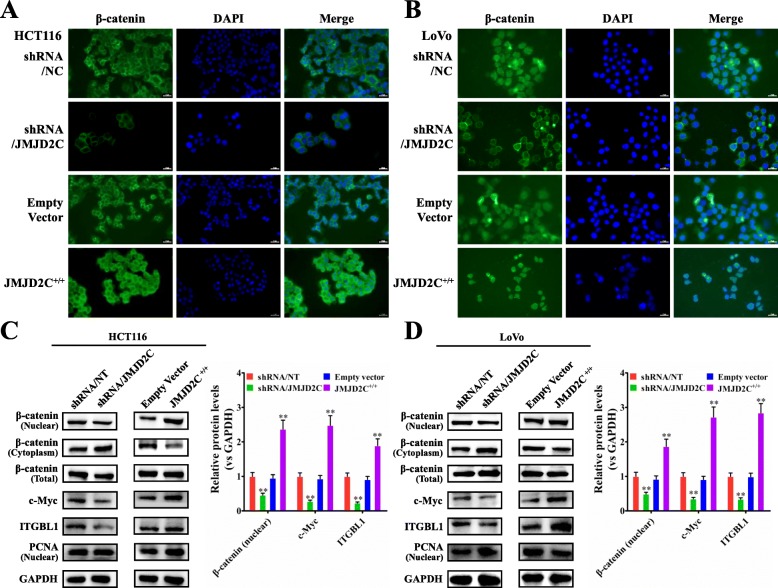
JMJD2C activated the β-catenin signaling pathway in CRC cells. **a**-**b** Immunofluorescence detection of β-catenin protein in HCT116 or LoVo cells transiently transfected with shRNA/NT vector, shRNA/JMJD2C vector, empty overexpression vector, or JMJD2C overexpression vector. **c**-**d** Western blot and quantitative assay of β-catenin protein (nuclear, cytoplasm and whole cell lysates) and β-catenin signaling downstream targets including c-Myc and ITGBL1 in HCT116 or LoVo cells transiently transfected with shRNA/NT vector, shRNA/JMJD2C vector, empty overexpression vector, or JMJD2C overexpression vector. *, *P* < 0.05; **, *P* < 0.01 (*t* test)

Our previous studies have demonstrated that MALAT1 could enhance the β-catenin signaling pathway [6, 7]; thus we further investigated the role of JMJD2C on regulating the expressions of β-catenin signaling pathway related proteins including β-catenin, c-Myc, and ITGBL1 (integrin β-like 1). By immunofluorescent staining assay, we found that, knockdown of JMJD2C could significantly decrease the nuclear accumulation of β-catenin protein in CRC cells, while overexpression of JMJD2C could effectively reverse the distribution of β-catenin in the nuclei of CRC cells (Fig. 5a, b). The following western blot results further confirmed the immunofluorescent staining results (Fig. 5c, d).

As we know, the nuclear β-catenin and other transcription factors such as LEF, TCF and BCL9 will function together to trans-activate downstream target genes [30]. Therefore, the luciferase reporter assay was further performed to observe the effect of JMJD2C on the activity of the LEF/TCF promoter. As expected, the final results confirmed the promoting effect of JMJD2C on the activity of the LEF/TCF promoter (Additional file 3: Figure S3). In addition, in Fig. 5c, d we also showed that, JMJD2C could enhance the expression of β-catenin signaling downstream targets including c-Myc and ITGBL1. All together, above data demonstrated JMJD2C enhanced the activity of β-catenin signaling pathway in CRC.

### JMJD2C promoted CRC lung metastasis in vivo

**Fig. 6:**
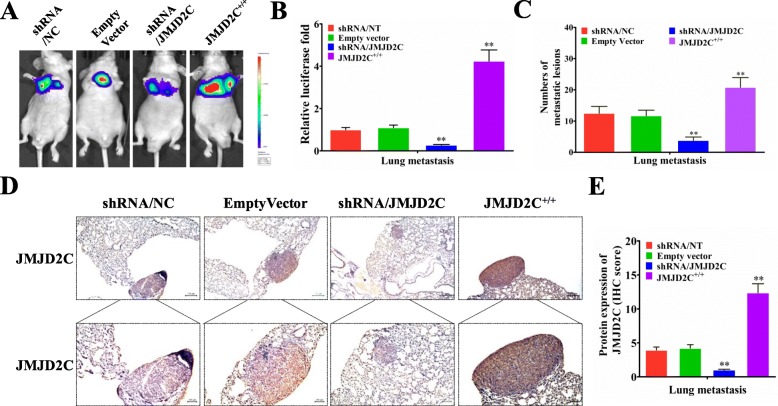
JMJD2C promoted the metastasis of CRC cells in vivo. **a-b** HCT116-shRNA/NT, HCT116-shRNA/JMJD2C, HCT116-empty vector, and HCT116-JMJD2C^+/+^ cells were respectively injected into the tail vein of nude mice (*n* = 6). After 42 days, the lung metastases images were observed and quantified by LB983 NIGHTOWL II system. **c** Quantification of lung metastatic nodules from 6 mice subjected to the indicated treatments. **d-e** Immunohistochemical and quantitative analysis of JMJD2C proteins on consecutive tissue microarray slides of lung metastatic nodules from 6 mice subjected to the indicated treatments. *, *P* < 0.05; **, *P* < 0.01 (*t* test)

In order to observe the in vivo effect of JMJD2C, HCT116-shRNA/NT, HCT116-shRNA/JMJD2C, HCT116-empty vector, and HCT116- JMJD2C^+/+^ cells were injected into nude mice (into the lateral tail vein), and the lung metastases images were observed by LB983 NIGHTOWL II (IVIS) system (Fig. 6a). As shown in Fig. 6b, knockdown of JMJD2C could significantly decrease the luciferase intensity of CRC cells in lung metastasis, while overexpression of JMJD2C could increase the luciferase intensity of CRC cells in lung metastasis. Additionally, the numbers of lung metastatic lesions were in accordance with the luciferase detecting results (Fig. 6c). Then, we detected the expression of JMJD2C protein in the lung metastatic lesions by immunohistochemical assay. The results showed the positive roles of JMJD2C in promoting CRC cells metastasis in vivo (Fig. 6d, e).

### JMJD2C elevated the expression of MALAT1 and β-catenin signaling related proteins in CRC lung metastasis mice models

**Fig. 7:**
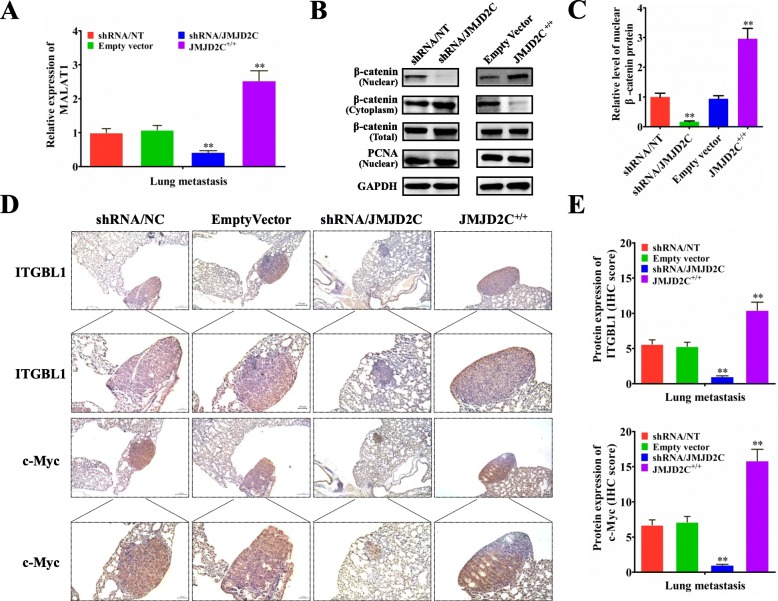
JMJD2C elevated the expression of MALAT1 and β-catenin signaling related proteins in CRC lung metastasis mice models. **a** Real-time PCR was performed to detect the expression of MALAT1 in lung metastatic nodules from 6 mice subjected to the indicated treatments. **b-c** Western blot and quantitative assay of β-catenin protein (nuclear, cytoplasm and whole cell lysates) in the lung metastatic tissues from 6 mice subjected to the indicated treatments. **d-e** Immunohistochemical and quantitative analysis of ITGBL1 and c-Myc proteins on consecutive tissue microarray slides of lung metastatic nodules from 6 mice subjected to the indicated treatments.*, *P* < 0.05; **, *P* < 0.01 (*t* test)

Since the in vitro study has found the regulatory effect of JMJD2C on the expression of MALAT1 and β-catenin signaling related proteins, we next validated the results in vivo. Real-time PCR showed that, knockdown of JMJD2C could significantly decrease the expression of MALAT1 in the lung metastatic lesions, while overexpression of JMJD2C could significantly elevate the expression of MALAT1 in the lung metastatic lesions (Fig. 7a). Then, the western blot experiments further demonstrated that, knockdown of JMJD2C could significantly decrease the nuclear accumulation of β-catenin protein in the lung metastatic lesions, while overexpression of JMJD2C could effectively reverse the distribution of β-catenin in the nuclei of lung metastatic lesions (Fig. 7b, c). By immunohistochemical assay we further found that, knockdown of JMJD2C could significantly decrease the expression of β-catenin signaling downstream targets including c-Myc and ITGBL1 in the lung metastatic lesions, while overexpression of JMJD2C could significantly elevate the expression of c-Myc and ITGBL1 in the lung metastatic lesions (Fig. 7d, e).

## Discussion

Histone demethylase JMJD2C has been reported to play crucial roles in the progression of colon cancer [19, 20], breast cancer [21–23], prostate cancer [24, 25], gastric cancer [26], lung cancer [27] and so on, indicating that JMJD2C represents a promising anti-cancer target.

JMJD2C is mapped to human chromosome 9p24.1, and it has been proved to be a demethylase for H3K9 and H3K36 methylation [28, 29]. More evidences have shown that JMJD2C is a candidate oncogene both in normal biology and tumorigenic processes [31]. Ye et al [23] found that knockdown of JMJD2C inhibited the proliferation of breast cancer cells in vitro and in vivo. In addition, Kim et al firstly uncovered that JMJD2C was overexpressed in five colon cancer cell lines and especially associated closely with the growth of HCT116 cells, and JMJD2C might promote the survival of colon cancer cells and stimulate the proliferation of colon cancer cells via up-regulating the levels of Cyclin D1 and FRA1 [19]. These results revealed the important roles of JMJD2C in tumors through targeting different oncogenes or tumor suppressor genes.

Our previous studies have successfully demonstrated that MALAT1 could promote CRC metastasis through regulating β-catenin signaling pathway [6, 7]. However, the upstream regulatory mechanism of MALAT1 is not well-elucidated. Based on bioinformatics analysis of the MALAT1 promoter, we found the potential binding sites for JMJD2C in the promoter of MALAT1, and hypothesized that, JMJD2C, the important histone demethylase, could influence the activity of MALAT1 promoter, thereby regulating MALAT1/β-catenin signaling pathway and leading to the promotion of CRC metastasis.

In the presented paper, we demonstrated that the mRNA expression levels of JMJD2C in CRC metastatic lesions are significantly higher than the ones in primary lesions, and there is a positive correlation between JMJD2C mRNA expression and MALAT1 expression. Our previous studies have shown the promoting effect of MALAT1 on the proliferation and migration of CRC cells [6]. Currently, our results further suggested that JMJD2C can also promote the proliferation and migration of CRC cells in vitro. Nevertheless, the direct evidence about the interaction between JMJD2C and MALAT1 is not presented. Various studies have demonstrated the complicated biological function of JMJD2C. Ishimura et al has proved that overexpression of JMJD2C could up-regulate the expression of oncogene Mdm2 and lead to the decreased expression of tumor suppressor gene p53 [32]. Our data suggested that JMJD2C elevated the expression of MALAT1 and regulated the β-catenin signaling pathway, as well as the downstream gene expression including c-Myc and ITGBL1. Further mechanism studies demonstrated that, JMJD2C could directly bind to the promoter region of MALAT1, lower the histone methylation level of MALAT1 promoter in the sites of H3K9me3 and H3K36me3, and enhance the promoter activity and the final expression of MALAT1.

Ye et al has shown that knockdown of JMJD2C not only inhibited the breast tumor growth but also effectively blocked the lung metastasis in mice model [23]. In our study, by establishing the lung metastasis mice model, we found that JMJD2C could promote CRC metastasis in vivo. Moreover, in vivo, we also found that, JMJD2C could significantly elevate the expression of MALAT1 in the lung metastatic lesions, increase the nuclear accumulation of β-catenin protein, and elevate the protein expression of downstream targets including c-Myc and ITGBL1 in the lung metastatic lesions. These results further confirmed that JMJD2C could up-regulate the MALAT1 expression and enhance the activity of β-catenin signaling pathway, which were in accordance with the results in vitro.

## Conclusion

**Fig. 8:**
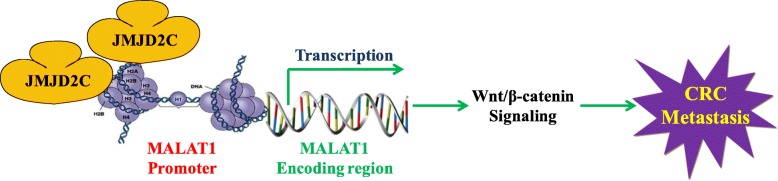
A schematic model of JMJD2C promoted CRC metastasis by regulating the histone methylation level of the MALAT1 promoter and enhancing β-catenin signaling pathway

In summary, our findings imply that JMJD2C plays a vital role in the epigenetic regulation process. In details, JMJD2C could directly influence the expression of MALAT1 by regulating the histone methylation level of MALAT1 promoter in the sites of H3K9me3 and H3K36me3, enhancing the activity of β-catenin signaling pathway, and promoting CRC metastasis (Fig. 8). Thus, JMJD2C might be a useful target for the prevention and treatment of CRC metastasis, and the interaction between JMJD2C and MALAT1 could be considered as the points for drug development.

## Supplementary information

**Additional file 1: Figure S1.** JMJD2C promoted the metastasis of CRC LoVo cells. a-c Real time PCR and western blotting were performed to confirm the gene silencing and overexpressing efficiency for JMJD2C. LoVo was transiently transfected with shRNA/NT vector, shRNA/JMJD2C vector, empty overexpression vector, or JMJD2C overexpression vector. d Migration assays of LoVo cells transfected with shRNA/NT, shRNA/JMJD2C, empty vector, or JMJD2C overexpression vector, respectively. e Numbers of migrated cells are shown as mean ± SD; *n* = 3. *, *P* < 0.05; **, *P* < 0.01 (*t* test).

**Additional file 2: Figure S2.** JMJD2C promoted the proliferation of CRC HCT116 cells, but affected little on the apoptosis of the indicated cells. a MTT assay of HCT116 cells transfected with shRNA/NT, shRNA/JMJD2C, empty vector, or JMJD2C overexpression vector, respectively. b Flow cytometry was performed to measure the apoptosis rates of HCT116 cells transfected with shRNA/NT, shRNA/JMJD2C, empty vector, or JMJD2C overexpression vector, respectively.

**Additional file 3: Figure S3.** JMJD2C enhanced the activity of the LEF/TCF promoter. a-b LEF/TCF promoter activity assay in HCT116 and LoVo cells transfected with shRNA/NT, shRNA/JMJD2C, empty vector, or JMJD2C overexpression vector, respectively. *, *P* < 0.05; **, *P* < 0.01 (*t* test).

## Data Availability

All of the data and materials in this paper are available when requested.

## Abbreviations

ChIP: Chromatin immunoprecipitation;
CRC: Colorectal cancer
DAB: Diaminobenzidine
HE: Hematoxylin and eosin;
IHC: Immunohistochemistry
ITGBL1: Integrin β-like 1
JMJD2C: Jumonji domain-containing protein 2C
lncRNAs: Long non-coding RNAs
MALAT1: Metastasis associated in lung adenocarcinoma transcript 1
OS: Overall survival
PTBP-2: Polypyrimidine tract binding protein
SFPQ: PTB-associated splicing factor
