# Correction: JMJD2C promotes colorectal cancer metastasis via regulating histone methylation of MALAT1 promoter and enhancing β-catenin signaling pathway

**DOI:** 10.1186/s13046-022-02407-0

**Published:** 2022-06-17

**Authors:** Xinnan Wu, Ruixiao Li, Qing Song, Chengcheng Zhang, Ru Jia, Zhifen Han, Lihong Zhou, Hua Sui, Xuan Liu, Huirong Zhu, Liu Yang, Yan Wang, Qing Ji, Qi Li

**Affiliations:** 1 Department of Medical Oncology and Cancer Institute of Medicine, Shuguang Hospital, Shanghai University of Traditional Chinese Medicine, Shanghai, China; 2 Department of Medical Oncology, Suzhou TCM Hospital Affiliated to Nanjing University of Chinese Medicine, Suzhou, China; 3 Department of Medical Oncology, Longhua Hospital, Shanghai University of Traditional Chinese Medicine, Shanghai, China; 4 Academy of Integrative Medicine, Shanghai University of Traditional Chinese Medicine, Shanghai, China

**Correction: J Exp Clin Cancer Res 38, 435 (2019)**

**https://doi.org/10.1186/s13046-019-1439-x**

Following publication of the original article [1], errors were identified in Figs. 2, 3, 7 and S1; specifically:

- Figure 2F: an image for the shRNA/JMJD2C group (72h) was incorrectly used for a representative picture; the correct image is now used; correspondingly, the quantitative graph in Fig. 2G has also been corrected
- Figure 3B: one set of immunofluorescence pictures for shRNA/NC group were incorrectly used for the representative pictures; the correct images are now used
- Figure 7: the order of shRNA/NC group and EmptyVector group for c-Myc was accidentally reversed in typesetting, which was inconsistent with the JMJD2C images in Figure 6 and ITGBL1 images in Figure 7; both sets of images have now been transposed to correct the error
- Figure S1D: an image for Empty Vector group was incorrectly used for a representative picture; the correct image is now used; correspondingly, the quantitative graph in Figure S1E has also been corrected.

The corrections do not have any effect on the final conclusions of the paper.

**Fig. 2:**
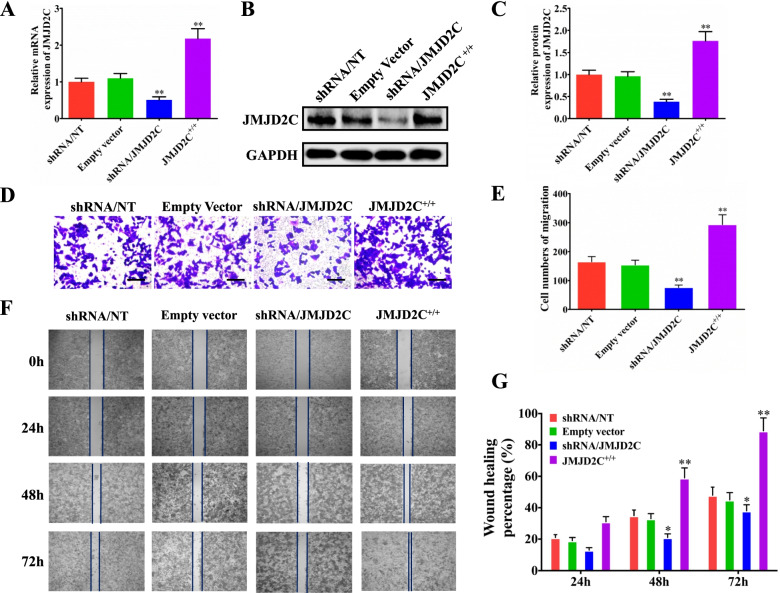
JMJD2C promoted the metastasis of CRC cells in vitro. **a-c** Real time PCR and western blotting were performed to confirm the gene silencing and overexpressing efficiency for JMJD2C. HCT116 was transiently transfected with shRNA/NT vector, shRNA/JMJD2C vector, empty overexpression vector, or JMJD2C overexpression vector. **d** Migration assays of HCT116 cells transfected with shRNA/NT, shRNA/JMJD2C, empty vector, or JMJD2C overexpression vector, respectively. **e** Numbers of migrated cells are shown as mean ± SD; *n* = 3. **f-g** Wound healing assay was used to evaluate the effect of JMJD2C on migration of HCT116 cells. *, *P* < 0.05; **, *P* < 0.01 (*t* test)

**Fig. 3:**
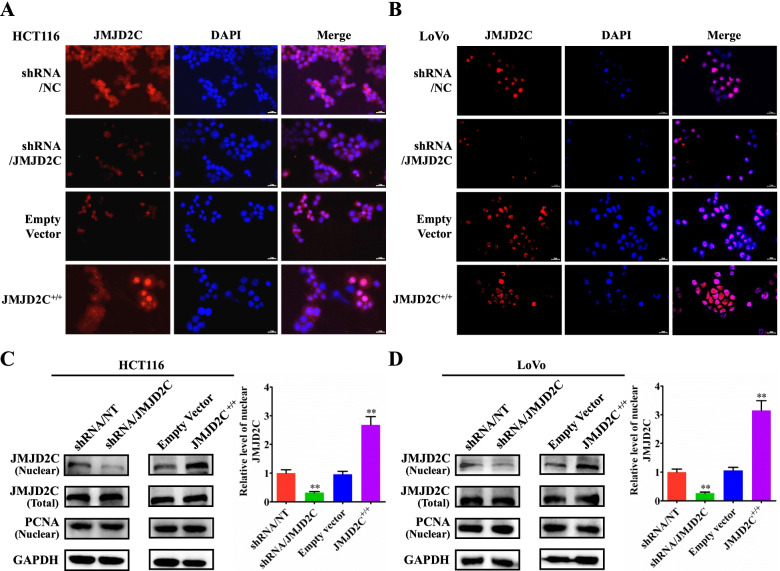
Translocation of JMJD2C protein from the cytoplasm into the nuclei in CRC cells in vitro. **a-b** Immunofluorescence detection of JMJD2Cprotein in HCT116 or LoVo cells transiently transfected with shRNA/NT vector, shRNA/JMJD2C vector, empty overexpression vector, or JMJD2C overexpression vector. **c-d** Western blot and quantitative assay of JMJD2C protein (nuclear and whole cell lysates) in HCT116 or LoVo cells transiently transfected with shRNA/NT vector, shRNA/JMJD2C vector, empty overexpression vector, or JMJD2C overexpression vector. *, *P* < 0.05; **, *P* < 0.01 (*t* test)

**Fig. 7:**
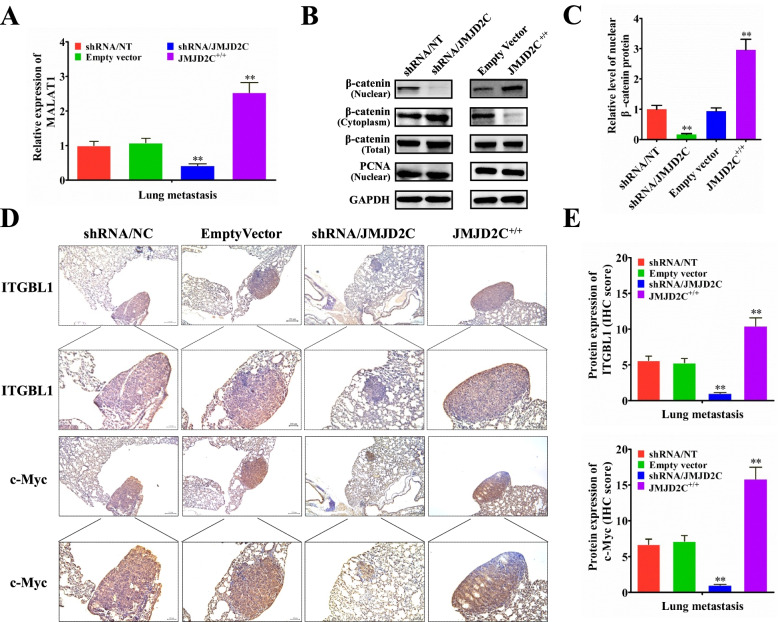
JMJD2C elevated the expression of MALAT1 and β-catenin signaling related proteins in CRC lung metastasis mice models. **a** Real-time PCR was performed to detect the expression of MALAT1 in lung metastatic nodules from 6 mice subjected to the indicated treatments. **b**-**c** Western blot and quantitative assay of β-catenin protein (nuclear, cytoplasm and whole cell lysates) in the lung metastatic tissues from 6 mice subjected to the indicated treatments. **d**-**e** Immunohistochemical and quantitative analysis of ITGBL1 and c-Myc proteins on consecutive tissue microarray slides of lung metastatic nodules from 6 mice subjected to the indicated treatments.*, *P* < 0.05; **, *P* < 0.01 (*t* test)

## Supplementary Information

**Additional file 1: Figure S1.** JMJD2C promoted the metastasis of CRC LoVo cells. a-c Real time PCR and western blotting were performed to confirm the gene silencing and overexpressing efficiency for JMJD2C. LoVo was transiently transfected with shRNA/NT vector, shRNA/JMJD2C vector, empty overexpression vector, or JMJD2C overexpression vector. d Migration assays of LoVo cells transfected with shRNA/NT, shRNA/ JMJD2C, empty vector, or JMJD2C overexpression vector, respectively. e Numbers of migrated cells are shown as mean ± SD; *n* = 3. *, *P* < 0.05; **, *P* < 0.01 (*t* test).

## References

[1] Wu X, Li R, Song Q (2019). JMJD2C promotes colorectal cancer metastasis via regulating histone methylation of MALAT1 promoter and enhancing β-catenin signaling pathway. J Exp Clin Cancer Res.

