# Supplementary figures and images for: JMJD2C promotes colorectal cancer metastasis via regulating histone methylation of MALAT1 promoter and enhancing β-catenin signaling pathway
Source: J Exp Clin Cancer Res. 2019 Oct 29;38:435. doi: 10.1186/s13046-019-1439-x (PMC6819649; doi:10.1186/s13046-019-1439-x)

**A**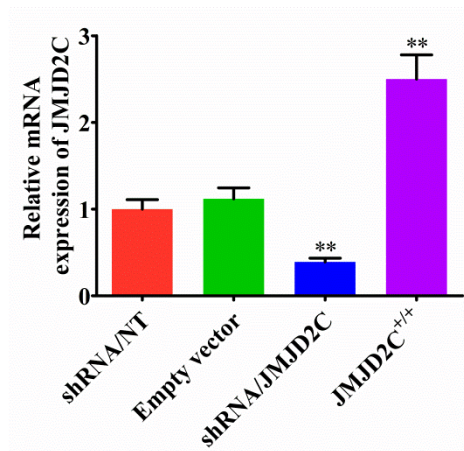**B**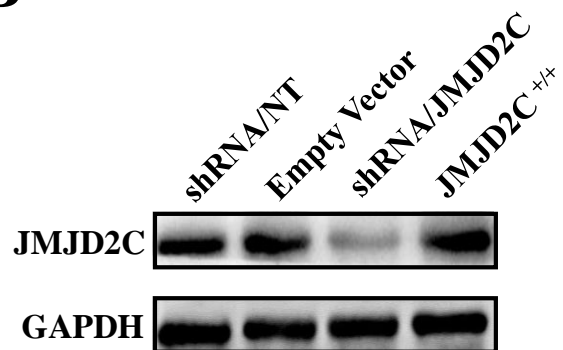**C**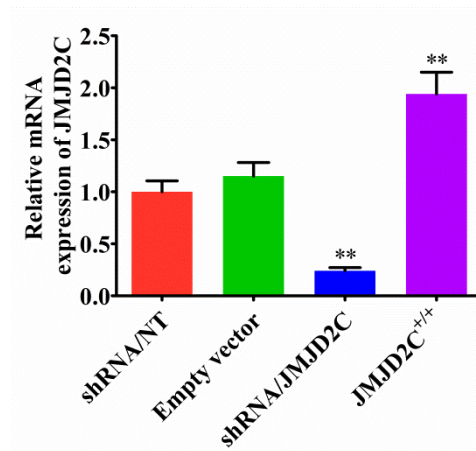**D**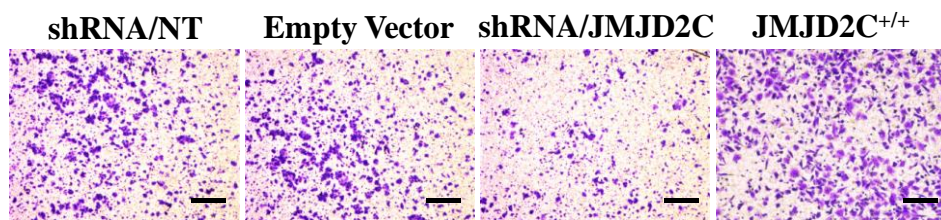**E**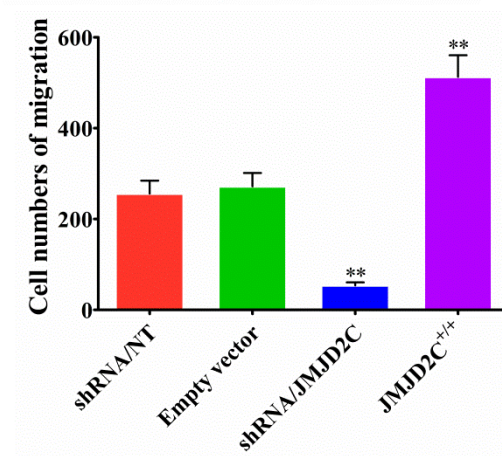

Supplement: Supplementary file 1 — Additional file 1: Figure S1. JMJD2C promoted the metastasis of CRC LoVo cells. a-c Real time PCR and western blotting were performed to confirm the gene silencing and overexpressing efficiency for JMJD2C. LoVo was transiently transfected with shRNA/NT vector, shRNA/JMJD2C vector, empty overexpression vector, or JMJD2C overexpression vector. d Migration assays of LoVo cells transfected with shRNA/NT, shRNA/JMJD2C, empty vector, or JMJD2C overexpression vector, respectively. e Numbers of migrated cells are shown as mean ± SD; n = 3. *, P < 0.05; **, P < 0.01 (t test). [file 13046_2019_1439_MOESM1_ESM.pdf]

**A**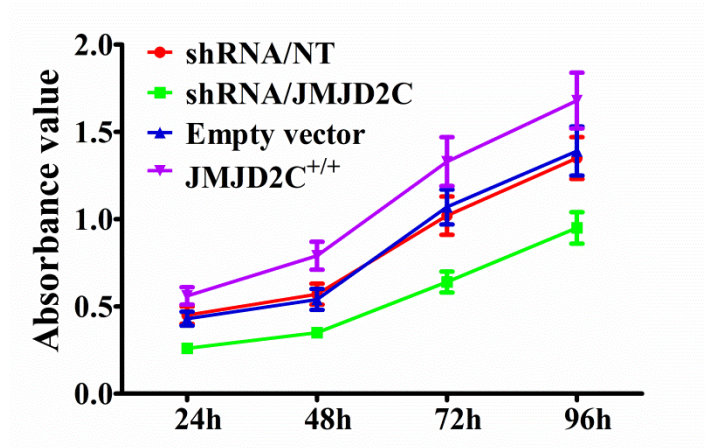**B**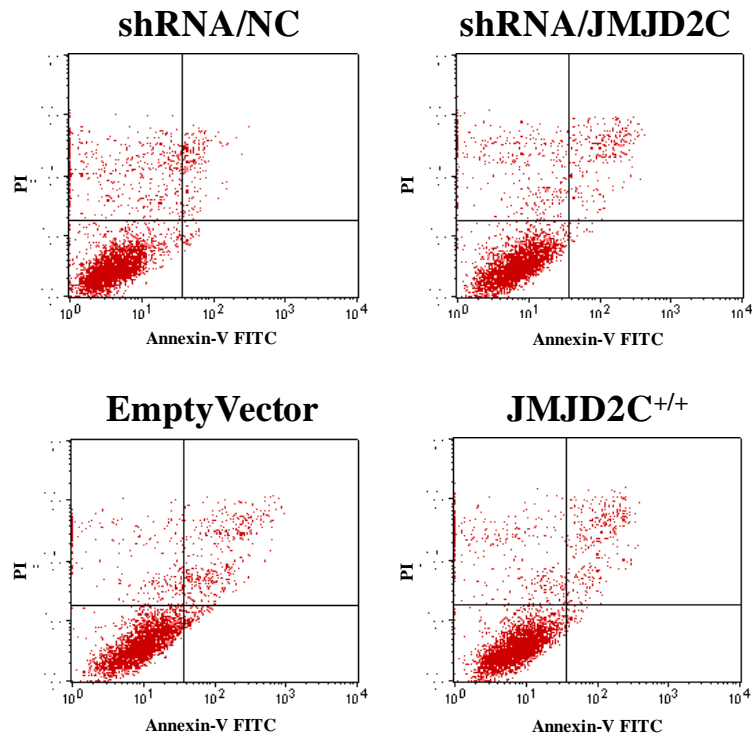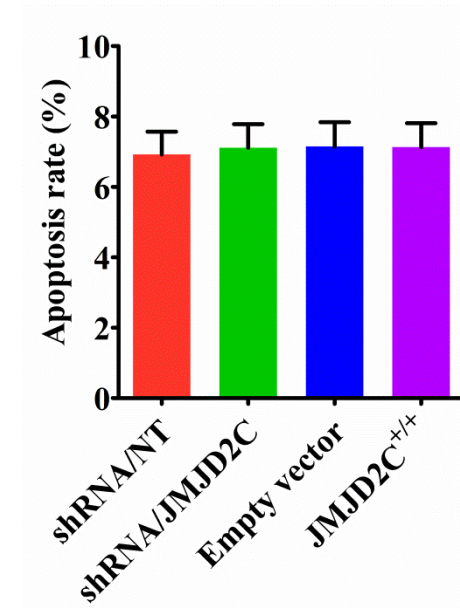

Supplement: Supplementary file 2 — Additional file 2: Figure S2. JMJD2C promoted the proliferation of CRC HCT116 cells, but affected little on the apoptosis of the indicated cells. a MTT assay of HCT116 cells transfected with shRNA/NT, shRNA/JMJD2C, empty vector, or JMJD2C overexpression vector, respectively. b Flow cytometry was performed to measure the apoptosis rates of HCT116 cells transfected with shRNA/NT, shRNA/JMJD2C, empty vector, or JMJD2C overexpression vector, respectively. [file 13046_2019_1439_MOESM2_ESM.pdf]

**A****HCT116**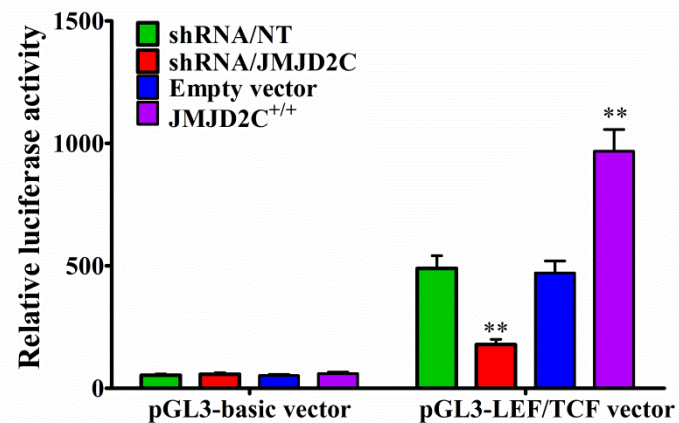**B****LoVo**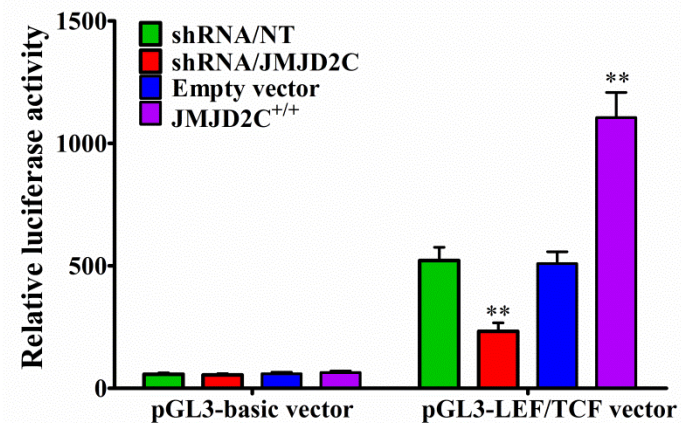

Supplement: Supplementary file 3 — Additional file 3: Figure S3. JMJD2C enhanced the activity of the LEF/TCF promoter. a-b LEF/TCF promoter activity assay in HCT116 and LoVo cells transfected with shRNA/NT, shRNA/JMJD2C, empty vector, or JMJD2C overexpression vector, respectively. *, P < 0.05; **, P < 0.01 (t test). [file 13046_2019_1439_MOESM3_ESM.pdf]
