# Supplementary figures and images for: Correction: JMJD2C promotes colorectal cancer metastasis via regulating histone methylation of MALAT1 promoter and enhancing β-catenin signaling pathway
Source: J Exp Clin Cancer Res. 2022 Jun 17;41:205. doi: 10.1186/s13046-022-02407-0 (PMC9205033; doi:10.1186/s13046-022-02407-0)

**A**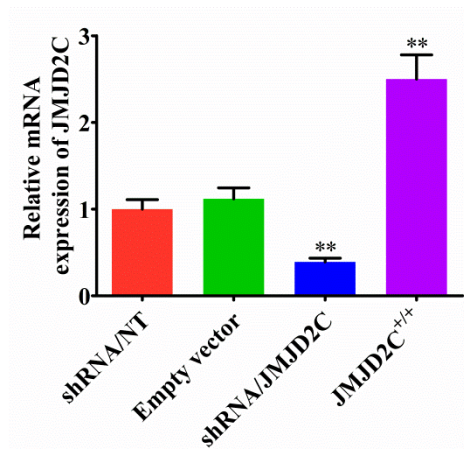**B**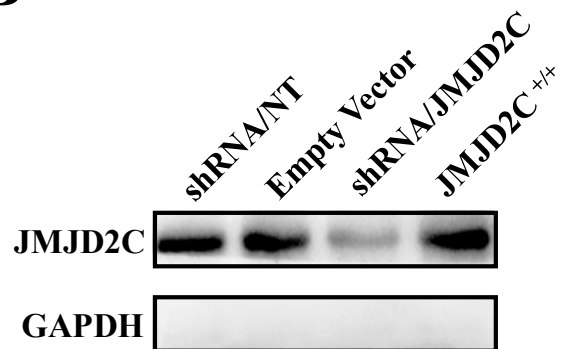**C**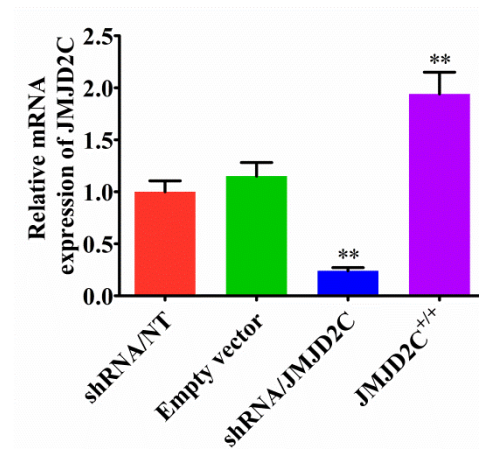**D**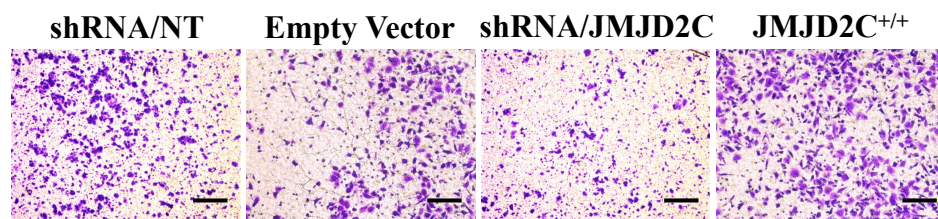**E**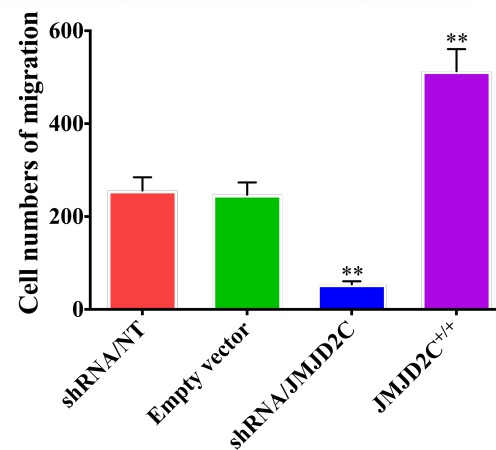

Supplement: Supplementary file 1 — Additional file 1: Figure S1. JMJD2C promoted the metastasis of CRC LoVo cells. a-c Real time PCR and western blotting were performed to confirm the gene silencing and overexpressing efficiency for JMJD2C. LoVo was transiently transfected with shRNA/NT vector, shRNA/JMJD2C vector, empty overexpression vector, or JMJD2C overexpression vector. d Migration assays of LoVo cells transfected with shRNA/NT, shRNA/ JMJD2C, empty vector, or JMJD2C overexpression vector, respectively. e Numbers of migrated cells are shown as mean ± SD; n = 3. *, P < 0.05; **, P < 0.01 (t test). [file 13046_2022_2407_MOESM1_ESM.pdf]
